# Sports Supplement Use in Road Cycling: A Comparative Analysis by Sex and Competitive Category †

**DOI:** 10.3390/sports13040122

**Published:** 2025-04-16

**Authors:** Jesús García-Durán, David Romero-García, José Miguel Martínez-Sanz, José Antonio González-Jurado, Antonio Jesús Sánchez-Oliver

**Affiliations:** 1 Faculty of Sports Science, Pablo de Olavide University, 41013 Seville, Spain; jgardur@alu.upo.es; 2 Research Center on Physical and Sports Performance, Pablo de Olavide University, 41013 Seville, Spain; sanchezoliver@us.es; 3 Optics, Pharmacology and Anatomy Department, Faculty of Sciences, University of Alicante, 03690 Alicante, Spain; david.romero@gcloud.ua.es; 4 Nursing Department, Faculty of Health Sciences, University of Alicante, 03690 Alicante, Spain; josemiguel.ms@ua.es; 5 Departamento de Motricidad Humana y Rendimiento Deportivo, Universidad de Sevilla, 41013 Seville, Spain

**Keywords:** endurance performance, sport nutrition, road cycling, ergogenic aids, doping

## Abstract

This study analyzes and compares sports supplement (SS) consumption among federated road cyclists, considering sex and competition category. The aim is to identify key factors influencing SS use and provide insights for developing nutritional strategies in cycling. A cross-sectional, descriptive study was conducted, involving 1503 cyclists (1231 men and 272 women). Data were collected through a validated questionnaire assessing anthropometric data, training habits, SS consumption patterns, and sources of information. Results indicate that 64.3% of cyclists currently use SS. Women reported a significantly higher consumption rate (88.2%) compared to men (59.1%), although men had a higher average SS intake than women (8.28 ± 9.36 vs. 6.76 ± 5.96). Additionally, SS use decreased with age and competition level, with elite cyclists showing the highest prevalence (76.3%) and master 50 the lowest (58.4%). Group A supplements (scientifically supported) were the most frequently used, while Group C supplements (limited evidence) and Group D substances (prohibited) were more commonly consumed by men. Findings highlight significant differences in SS consumption based on sex and competition level, with elite cyclists and women reporting higher prevalence. However, men reported a higher average number of SS consumed. The study underscores the need for targeted nutritional education, particularly among master cyclists, to promote evidence-based SS use and minimize the risks of ineffective or unsafe supplementation. Future research should explore the long-term effects of SS consumption in cycling and the effectiveness of educational interventions for safe and optimized supplementation practices.

## 1. Introduction

Road cycling has experienced significant growth [1,2], characterized by high physical and metabolic demands, particularly in aerobic capacity and lactate threshold [3,4]. These factors, alongside biomechanical and psychological aspects, are key to competitive success [5,6], making nutritional planning essential for optimizing performance and recovery [7,8].

One of the most relevant elements in cyclists’ nutritional planning is the consumption of sports supplements (SSs), which have been widely used to improve energy and macronutrient availability, optimize performance, and prevent nutritional deficiencies [8,9]. Excessive or misguided supplementation can lead to financial waste, ineffective performance enhancement, or even adverse health and sport performance effects [10,11].

The Australian Institute of Sport (AIS) categorizes SS into four groups based on scientific evidence: Group A (strong scientific support), Group B (emerging evidence), Group C (limited evidence), and Group D (prohibited or contaminant substances) [9]. While Group A supplements are widely recommended, the inappropriate use of substances from Groups C and D raises concerns regarding efficacy, safety, and the risk of unintentional doping [9]. Despite the availability of scientifically backed supplements, the misuse of banned substances has been observed, with a high prevalence among federated road cyclists [12].

SSs are often used inappropriately due to concerns about their safety, efficacy, and legality, as well as their consumption in unsuitable situations [13]. To ensure responsible use, supplementation should be justified based on specific needs and adapted to the athlete’s age and life cycle [14]. Key reasons for SS use include correcting nutrient deficiencies, addressing inadequate energy intake, supporting restrictive diets, managing food allergies or intolerances, optimizing training adaptations, enhancing performance, and ensuring adequate nutrition during travel to areas with food availability or safety concerns [15].

The scarce existing literature suggests that SS consumption varies by sex and competition level among cyclists. Studies indicate that female cyclists report a higher prevalence of SS use than their male counterparts [12,16], though men often consume a greater number of supplements [8,12,16,17]. Additionally, competitive category influences SS intake, with elite athletes showing a higher reliance on evidence-based ergogenic aids, while lower-category cyclists may be more prone to using supplements with limited scientific backing [12,16]. Understanding these patterns is essential for developing targeted nutritional education and policies to promote safe and effective supplementation practices and to address the distinct needs of male and female cyclists at different levels of competition [18,19].

To date, no study has specifically examined a sample of federated road cyclists to compare SS consumption by sex and competition category. The present study aims to analyze and compare SS consumption among federated road cyclists, with a focus on sex and competition category. By addressing existing gaps in the literature, this study seeks to provide insights into the factors influencing SS use in cycling, contributing to evidence-based recommendations for optimizing nutritional strategies in cycling.

## 2. Materials and Methods

### 2.1. Study Design and Participants

This study follows a quantitative, cross-sectional, and descriptive design, focusing on the analysis of SS consumption among federated road cyclists. Participant selection was conducted through the Andalusian Cycling Federation, as well as cycling clubs and sports associations in the region. A non-probabilistic sampling method was employed, and the final sample included cyclists of both sexes, distributed across various competition categories: elite, master 30, master 40, and master 50. Table 1 presents descriptive data on the sample, including age, basic anthropometric characteristics, and sports-related aspects, segmented by sex and category.

The sample size was determined using the Australian Bureau of Statistics (https://www.abs.gov.au/websitedbs/D3310114.nsf/home/Sample+Size+Calculator, accessed on 15 January 2025) Sample Size Calculator, with a 95% confidence level. According to FAC data from 2023, the target population consisted of 8189 federated cyclists (371 women and 7818 men). Assuming an expected proportion of 0.5, the final sample included 1503 cyclists (men: *n* = 1231; women: *n* = 272), with a confidence interval of 0.02, a standard error of 0.01, and a relative standard error of 2.33.

### 2.2. Data Collection Instrument

For data collection, a previously validated questionnaire was used and underwent a content, structure, and applicability review [20]. The questionnaire consisted of three main sections: (a) anthropometric and personal data (age, weight, height, years of federation, weekly training days and hours, number of competitions per year); (b) sports practice, including information on competition category and experience level; (c) SS consumption habits, which assessed types of supplements used, reasons for consumption, sources of information, purchasing locations, consumption frequency, and perceived results [21].

The questionnaire used in this study was reviewed and classified among the validated instruments included in the systematic review and meta-analysis conducted by Knapik et al. (2016), which assessed its adequacy for obtaining accurate data on SS use among athlete populations [17].

### 2.3. Procedure

Participants were contacted through the FAC and sports clubs via email, where they received detailed study information and a link to the questionnaire. Additionally, data collection was conducted in person at various competitions held between January and May 2024. For the in-person data collection, we used tablets to administer the questionnaire via Google Forms, with responses being automatically recorded in a Google Sheet.

Participation was voluntary and anonymous, ensuring compliance with the principles of the Declaration of Helsinki for research involving human subjects. Before participating, all subjects in the study were fully informed and consented. The study protocol was approved by the Ethics Committee of the Pablo de Olavide University (reference number: 27/7-3).

### 2.4. Statistical Analysis

To assess variable distribution and homoscedasticity, the Kolmogorov–Smirnov and Levene tests were applied. After confirming a normal distribution, a descriptive analysis was performed, presenting mean and standard deviation (Mean ± SD).

Differences in SS consumption by sex (women vs. men), based on the categories established by the AIS [9], were analyzed using Student’s *t*-test, and the effect size was calculated using Cohen’s d coefficient. To examine differences across competition categories (elite, master 30, master 40, and master 50), a one-way ANOVA was conducted, with effect size calculated using partial eta squared (ηp^2^). Subsequently, Bonferroni-adjusted pairwise comparisons were performed for variables showing significant differences. The level of significance was set a priori at *p* < 0.05.

For qualitative variables, including current SS consumption and supplements used by more than 10% of the sample, a chi-square test (X^2^) was applied to assess potential differences based on sex and competition category. Residuals and Cramér’s V were also calculated to evaluate the association between different groups. The level of significance was set a priori at *p* < 0.05. All statistical analyses were conducted using the Statistical Package for Social Sciences (SPSS) v.25 (IBM, Armonk, NY, USA).

## 3. Results

Of the total sample, 64.3% reported currently consuming some type of SS. Regarding sex, significant differences were found between women and men (*p* < 0.001), with women showing a higher consumption rate (88.2% vs. 59.1%). Regarding the competition category, significant differences were also observed (*p* < 0.001), indicating that SS consumption decreased as the age category increased, with elite cyclists reporting the highest consumption (76.3%, 64.2%, 60.8%, 58.4%).

Table 2 shows that men consumed significantly more total SS, Group A ergogenic aids, and Group C and D supplements than women. When looking at competition categories, there were significant differences in consumption for all analyzed variables.

Post hoc pairwise comparisons between different categories, adjusted using the Bonferroni correction and considering variables with significant differences by competition level, revealed significant differences between elite cyclists and all other categories (*p* < 0.001–0.008) for most variables analyzed, with elite cyclists consistently showing the highest SS consumption. The only exception was observed in Group D, where no significant differences were found when comparing elite cyclists with the master 30 category. Additionally, no significant differences were found when comparing elite and master 30 cyclists in terms of total SS consumption (*p* = 0.215).

When comparing master 30 with master 40 and master 50, significant differences were found in most analyzed variables (*p* < 0.001–0.037), with master 30 cyclists showing higher consumption levels. However, no significant differences were observed when comparing master 30 vs. master 50 in medical supplements (*p* = 0.174), master 30 vs. master 40 and 50 in Group B supplements (*p* = 1.000), or master 30 vs. master 40 in Group C supplements (*p* = 0.499). Finally, when comparing master 40 and master 50, significant differences were found in total SS consumption, ergogenic aids from Group A, and Group C supplements (*p* < 0.001–0.037), with higher consumption in master 40 cyclists. No significant differences were observed for the remaining variables (*p* = 0.294–1.000).

The post hoc analysis also showed that elite cyclists reported significantly higher SS consumption compared to master categories, especially when compared to master 40 and master 50 (*p* < 0.001). In Group A, elite cyclists consumed more sports foods, medical supplements, and ergogenic aids compared to master categories. In particular, ergogenic aids were more frequently used by elite cyclists than by those in master 40 and master 50 categories (*p* < 0.001), whereas differences between master 30 and master 40 were smaller.

In Group B, significant differences were found between elite cyclists and master categories in most cases, with lower consumption levels as age increased. A similar pattern was observed in Group C, where elite cyclists used more supplements with little scientific evidence compared to master cyclists. Finally, in Group D (prohibited or high-risk substances), higher consumption was identified in the master 30 category compared to other categories, highlighting the need for greater control and nutritional education in these groups.

Table 3 and Table 4 present the SS consumed by at least 10% of the total sample, segmented by sex and competition level. Regarding sex, significant differences were observed for most SS analyzed, except for gels, iron, multivitamins, probiotics, beta-alanine, or glutamine. Regarding competition categories, significant differences were found for most analyzed SS, except for melatonin. SS consumption decreased as age categories increased, with elite cyclists reporting the highest intake levels. The consumption of sports drinks, sports bars, electrolytes, caffeine, beta-alanine, creatine, nitrates, BCAAs, mineral complexes, and spirulina showed a progressive decline across older categories, with elite cyclists consuming the most. Carbohydrate gainers, gels, iron, vitamin C, and citrulline malate were significantly more consumed in the elite category. Casein and multivitamins were more frequently consumed by elite and master 30 cyclists. Probiotics were consumed more frequently by the elite and master 50 categories. Finally, vitamin D, magnesium, and glutamine had a higher consumption rate in the master 40 category.

## 4. Discussion

A total of 64.3% of cyclists reported consuming SS, with a higher prevalence among women and elite athletes. While Group A supplements were the most commonly used, a significant consumption of Group C (limited scientific evidence) and Group D (prohibited substances) supplements was also observed, particularly among men.

This study reveals a high percentage of SS consumption among federated road cyclists, with significant differences based on sex and competition category. Overall, 64.3% of the sample reported SS use. These results are significantly lower than those reported in other studies, such as the 100% recorded among Canadian Olympic cyclists during the Sydney Olympics [22], 97.5% among elite U-23 male cyclists [23], and 85% among high-level Spanish cyclists [24]. This variation may be attributed to differences in the competitive level of these cyclists compared to the present sample, in which only a portion competed in the elite category. Regarding sex, there was a notably higher prevalence among women (88.2%) compared to men (59.1%). This finding contrasts with previous studies indicating higher SS consumption among men [7,17,25]. While research on supplement use in female cyclists is scarce, this is also evident in studies conducted on cyclists, where men had higher SS consumption (85–100%) [22,23,24] than women (85.1%) [16]. However, recent research suggests an increasing trend of supplement use among female athletes [16], potentially due to greater awareness of their specific nutritional needs and improved access to specialized guidance [18,19]. Additionally, the growing professionalization of women’s cycling may be facilitating more equitable access to advanced nutritional strategies [1].

The number of SSs consumed also showed significant differences based on sex and competition category. On average, men consumed more supplements than women (8.3 ± 9.4 vs. 6.8 ± 6.0), suggesting a broader variety of products integrated into their nutritional strategies. This discrepancy may stem from marketing influence, the widespread supplement culture in male sports, and the perception that certain products provide competitive advantages [13,14]. In contrast, women’s supplement choices were more focused on preventing nutritional deficiencies and optimizing recovery, with higher consumption of iron and vitamin D. These patterns align with physiological differences, such as the higher risk of deficiencies in female athletes due to menstruation and other factors, and highlight a greater reliance on specialized guidance among women [18,19].

Regarding competition level, SS consumption was highest among elite cyclists (76.3%) and progressively declined with increasing age and lower competition levels. This trend aligns with previous studies in endurance sports [17,26,27,28]. This finding could be that elite athletes have greater access to nutritional resources, structured dietary plans, and personalized supplementation strategies [8,25,29]. Conversely, master cyclists, particularly those in the master 40 and master 50 categories, reported significantly lower SS consumption, likely due to differences in performance priorities, reduced access to expert guidance, or lower susceptibility to the supplementation trends commonly observed at elite levels [12,16]. Total supplement consumption was highest among elite cyclists (11.3 ± 10.8 supplements), while older cyclists, particularly those in the master 50 category, exhibited lower usage (4.9 ± 5.0 supplements).

Regarding the high number of SS consumed by the sample, it should be emphasized that even evidence-based dietary products can cause side effects, particularly at high doses. Adverse effects often stem more from consumer and vendor practices than from the ingredients themselves. Additionally, interactions may arise when multiple products are taken simultaneously, posing potential risks, especially for athletes who consume various products daily, often in high doses and without full awareness of their combined effects. In extreme cases, excessive consumption has led to severe health consequences, highlighting the need for informed and responsible use [30].

In terms of supplement classification, Group A supplements, which have the highest scientific support, were the most widely used, consistent with findings from other endurance sports where scientifically validated sports foods, medical supplements, and ergogenic aids are predominant [26,27,28]. However, the study also revealed a considerable intake of Group C supplements, which lack conclusive efficacy, suggesting potential gaps in knowledge regarding their real benefits or the influence of external factors such as aggressive marketing [9,13]. Of particular concern is the presence of Group D supplements, albeit limited, which are prohibited or pose doping risks [9]. A significantly higher consumption of these substances was observed among men, potentially due to greater competitive pressure and a perception that these products offer performance benefits [31].

From an applied perspective, these findings highlight the need to develop specific nutritional education and supplementation strategies tailored to cyclists across different categories [14]. The high prevalence of Group C and D supplements underscores the importance of providing evidence-based information to prevent the use of ineffective or potentially harmful products [9]. Ensuring equitable access to specialized guidance is particularly crucial for master cyclists, who reported lower consumption of scientifically supported supplements. Moreover, even though most supplements consumed by the sample belonged to Group A, supported by scientific evidence, they did not utilize other supplements with demonstrated potential to enhance performance in endurance sports, such as sodium bicarbonate and glycerol [32,33]. In addition, collagen and omega-3 (e.g., fish oil) may be beneficial in the nutritional management of musculoskeletal injuries in athletes, but further research is required [34].

Despite sports supplements are commonly used by athletes, their implementation is frequently suboptimal and inadequate [13,15]. Therefore, sports organizations, sports science and medicine practitioners, coaches, and athletes must adopt a practical and transparent approach that weighs the advantages and disadvantages of supplement use by evaluating their safety, effectiveness, and permissibility in sports [9]. This highlights the importance of conducting cost–benefit analyses to evaluate their responsible use, combined with personalized nutritional assessments tailored to athletes’ specific contexts and requirements [14]. It is also critical to emphasize that supplements should not serve as a substitute for unbalanced diets or nutritional deficiencies [35]. Moreover, the excessive or unregulated consumption of these products poses serious risks, underscoring the need for educational initiatives starting at an early age to promote informed decision-making among athletes, coaches, and family members [15].

Although this study provides relevant information on SS consumption among federated cyclists according to competitive category and sex, several limitations must be considered when interpreting the results. First, although the study includes a large sample of federated cyclists across different competitive categories, data were collected at a single point in time. This limits the ability to extrapolate trends over the years, making it advisable to conduct longitudinal studies to analyze the evolution of SS consumption in this population. Second, the data are based on self-reported information obtained through a validated questionnaire, which may introduce biases in the reporting of supplement intake. Specifically, the low reported consumption of Group D substances could be influenced by participants being unwilling to disclose their use due to potential negative consequences or social stigma. Furthermore, individuals using such substances may have been less likely to participate in the survey altogether, leading to an underestimation of their prevalence. Finally, future research should explore the relationship between SS consumption and athletic performance in cyclists across different categories, which would allow for the identification of specific supplements and consumption patterns associated with various performance levels.

Future research should further investigate the underlying reasons for differences in SS consumption based on sex and competition level and explore the long-term impacts of supplementation on cycling performance and health. Evaluating the effectiveness of intervention strategies aimed at optimizing SS consumption in competitive cycling will be essential for promoting safe and effective nutritional practices. Additionally, future research should continue to monitor supplement consumption trends in road cycling, explore the motivations behind supplement choices, and evaluate the impact of educational interventions on athletes’ nutritional practices.

## 5. Conclusions

This study reveals that sports supplement consumption is high among federated road cyclists, with notable variations influenced by both sex and competitive level. While women exhibit a higher overall prevalence of supplement use, men tend to consume a wider array and greater quantities of supplements. Supplementation patterns are also strongly linked to competition category, with elite cyclists demonstrating the highest engagement, which gradually declines across the master categories. Although supplements with strong scientific backing (Group A) are the most frequently used, the significant consumption of supplements with limited evidence (Group C) and potentially harmful or prohibited substances (Group D), particularly among men, raises concerns. These findings highlight the complex interplay between sex, competitive demands, and supplementation behaviors in road cycling.

Based on these conclusions, several recommendations can be made. Firstly, there is a clear need for targeted nutritional education programs tailored to the specific needs and consumption patterns of road cyclists, considering both sex and competition category. These programs should emphasize the importance of evidence-based supplementation and aim to discourage the use of ineffective or potentially risky products. Secondly, efforts should be made to ensure qualified sports nutrition professionals for cyclists at all levels, particularly for who may have less access to such resources. Sports organizations and coaches should play a proactive role in promoting informed decision-making regarding supplement use, emphasizing that supplements should complement, not replace, a balanced diet. Finally, given the concerning use of Group C and D supplements, stricter regulations and increased awareness campaigns regarding the risks associated with these substances may be warranted within the cycling community.

## Figures and Tables

**Table 1:** Descriptive data of the sample by sex and competition category.

	Sex (M ± SD)		Category (M ± SD)			
	Women (*n* = 272)	Men (*n* = 1231)	Elite (*n* = 312)	Master 30 (*n* = 386)	Master 40 (*n* = 444)	Master 50 (*n* = 361)
Age (years)	40.2 ± 9.6	41.6 ± 9.9	29.3 ± 4.0	34.7 ± 2.9	44.6 ± 2.9	54.7 ± 3.0
Height (cm)	162.7 ± 7.0	171.1 ± 7.3	170.8 ± 7.7	171.0 ± 7.9	170.5 ± 8.9	166.0 ± 5.6
Body Mass (kg)	60.0 ± 7.3	65.0 ± 7.2	61.9 ± 6.9	63.9 ± 8.0	65.5 ± 8.3	64.5 ± 5.5
Years Licensed	7.7 ± 3.0	7.0 ± 3.1	6.2 ± 2.9	5.4 ± 2.9	7.6 ± 3.2	9.3 ± 1.8
Weekly Training Days	4.2 ± 1.1	4.4 ± 0.9	4.7 ± 0.9	4.4 ± 1.0	4.3 ± 0.8	4.5 ± 0.8
Daily Training Hours	2.4 ± 0.6	4.2 ± 1.2	4.8 ± 1.4	4.0 ± 1.2	3.6 ± 1.1	3.2 ± 0.9
No. Competitions in the Season	9.1 ± 3.1	9.8 ± 2.5	10.8 ± 1.9	10.5 ± 1.9	10.0 ± 2.1	7.4 ± 3.0

M: mean; SD: standard deviation.

**Table 2:** Number of SSs used based on the level of evidence established by the AIS [9], segmented by sex and competition category.

		Sex (M ± SD)					Categories (M ± SD)						
		Women	Men	t	*p*	d Cohen	Elite	M 30	M 40	M 50	F	*p*	ηp2
Total SS		6.8 ± 6.0	8.3 ± 9.4	−3.39	<0.001	8.85	11.3 ± 10.8	10.0 ± 10.0	6.5 ± 7.4	4.9 ± 5.0	43.63	<0.001	0.08
Group A	Total	4.8 ± 3.1	4.8 ± 4.7	−0.23	0.815	4.46	7.0 ± 5.4	5.1 ± 4.3	4.1 ± 4.1	3.5 ± 3.4	43.19	<0.001	0.08
	Sport foods	3.0 ± 1.6	2.9 ± 2.7	0.74	0.458	2.50	4.3 ± 2.9	3.0 ± 2.2	2.5 ± 2.4	2.2 ± 2.1	47.67	<0.001	0.09
	Medical Sup.	1.0 ± 1.3	1.0 ± 1.2	0.36	0.720	1.21	1.4 ± 1.3	1.0 ± 1.2	0.8 ± 1.1	0.8 ± 1.2	19.15	<0.001	0.04
	Ergogenic aids	0.8 ± 1.0	1.0 ± 1.3	−2.47	0.014	1.22	1.4 ± 1.5	1.1 ± 1.3	0.9 ± 1.2	0.5 ± 0.7	36.05	<0.001	0.07
Group B		0.4 ± 0.9	0.5 ± 0.9	−1.88	0.060	0.93	0.8 ± 1.2	0.4 ± 1.0	0.5 ± 0.8	0.4 ± 0.7	17.81	<0.001	0.03
Group C		1.6 ± 2.9	2.0 ± 3.4	−2.28	0.023	3.29	3.0 ± 4.1	2.2 ± 3.7	1.8 ± 3.0	0.9 ± 1.5	23.93	<0.001	0.05
Group D		0.0 ± 0.1	0.9 ± 1.7	−18.28	<0.001	1.55	0.5 ± 1.0	2.3 ± 2.3	0.2 ± 0.5	0.0 ± 0.2	248.12	<0.001	0.33

**Table 3:** Distribution (%) of the most consumed SS by sample, based on sex and according to the level of evidence established by the AIS [9].

Category			Total	Women	Residual	Men	Residual	X^2^	*p*	V Cramer
Group A	Sport foods	Sports bars	61.9	80.5	7.0	57.8	7.0	48.591	<0.001	0.180
		Sport gels	60.8	64.7	1.5	60.0	−1.5	2.113	0.146	0.037
		Sports drinks	53.4	73.9	7.5	48.8	−7.5	56.283	<0.001	0.194
		CG	44.1	27.2	−6.2	47.8	6.2	38.501	<0.001	0.160
		Electrolytes	36.1	46.0	3.8	33.9	−3.8	14.102	<0.001	0.097
		Casein	15.0	4.0	−5.6	17.5	5.6	31.411	<0.001	0.145
	Medical Supplements	Multivitamin	39.3	36.8	−1.0	39.9	1	0.910	0.340	0.025
		Iron	23.8	21.7	−0.9	24.2	0.9	0.779	0.377	0.023
		Vitamin D	15.3	19.5	2.1	14.4	−2.1	4.482	0.034	0.055
		Probiotics	17.3	15.4	−0.9	17.7	0.9	0.801	0.371	0.023
	Ergogenic aids	Caffeine	40.4	49.6	3.4	38.3	−3.4	11.793	<0.001	0.089
		Nitrates	16.2	3.3	−6.4	19.0	6.4	40.515	<0.001	0.164
		CM	16.0	4.4	−5.8	18.6	5.4	33.322	<0.001	0.149
		Beta-alanine	14.5	11.0	−1.8	15.3	1.8	3.234	0.072	0.046
Group B		Vitamin C	24.2	11.0	−5.6	27.1	5.6	31.476	<0.001	0.145
Group C		BCAA	36.2	29.4	−2.6	37.7	2.6	6.615	0.010	0.066
		Spirulina	13.1	1.8	−6.1	15.6	6.1	37.029	<0.001	0.157
		Mineral complex	13.0	5.5	−4.1	14.7	4.1	16.587	<0.001	0.105
		Melatonin	12.2	3.7	−4.7	14.1	4.7	22.435	<0.001	0.122
		Magnesium	12.1	23.2	6.2	9.7	−6.2	38.119	<0.001	0.159
		Citrulline Malate	11.6	0.0	−6.6	14.2	6.6	43.763	<0.001	0.171
		Glutamine	11.0	10.3	−0.4	11.1	0.4	0.159	0.690	0.010

CG: Carbohydrate gainers; CM: Creatine monohydrate; BCAA: Branched-Chain Amino Acids.

**Table 4:** Distribution (%) of the most consumed SS by sample, based on competition category and according to the level of evidence established by the AIS [9].

			Total	Elite	Residual	M-30	Residual	M-40	Residual	M-50	Residual	X^2^	*p*	V Cramer
Group A	Sport foods	Sports bars	61.9	76.6	6.0	62.4	0.2	59.5	−1.3	51.8	−4.6	45.397	<0.001	0.174
		Sport gels	60.8	74.7	5.6	63.7	1.4	53.8	−3.6	54.3	−2.9	42.079	<0.001	0.167
		Sports drinks	53.4	71.8	7.3	60.1	3.1	46.4	−3.5	38.8	−6.4	89.140	<0.001	0.244
		CG	44.1	59.0	5.9	44.8	0.3	30.9	−6.7	46.8	1.2	60.750	<0.001	0.201
		Electrolytes	36.1	59.6	9.7	39.1	1.5	28.8	−3.8	21.3	−6.7	120.690	<0.001	0.283
		Casein	15.0	21.8	3.8	21.8	4.3	13.1	−1.4	4.4	−6.5	57.949	<0.001	0.196
	Medical Suppl.	Multivitamin	39.3	64.1	10.1	52.8	6.3	14.0	−13.0	34.6	−2.1	232.902	<0.001	0.394
		Iron	23.8	34.0	4.8	22.3	−0.8	19.6	−2.5	21.6	−1.1	23.619	<0.001	0.125
		Vitamin D	15.3	18.3	1.6	10.6	−3.0	25.7	7.2	5.0	−6.2	75.148	<0.001	0.224
		Probiotics	17.3	21.8	2.4	15.0	−1.4	13.5	−2.5	20.5	1.8	12.833	0.005	0.092
	Ergogenic aids	Caffeine	40.4	51.3	4.4	43.3	1.3	39.2	−0.6	29.4	−4.9	35.197	<0.001	0.153
		Nitrates	16.2	26.3	5.5	21.8	3.5	13.3	−2.0	5.0	−6.6	68.477	<0.001	0.213
		Beta-alanine	14.5	28.5	7.9	16.1	1.0	13.1	−1.0	2.5	−7.4	92.963	<0.001	0.249
		CM	16.0	20.8	2.6	18.4	1.5	16.4	0.3	8.9	−4.3	20.772	<0.001	0.118
Group B		Vitamin C	24.2	24.2	6.7	13.7	−5.6	24.5	0.2	22.4	−0.9	59.841	<0.001	0.200
Group C		BCAA	36.2	36.2	7.7	42.2	2.9	26.8	−4.9	25.2	−5.0	88.720	<0.001	0.243
		Spirulina	13.1	13.1	2.5	16.3	2.2	14.2	0.8	4.7	−5.9	31.146	<0.001	0.144
		MC	13.0	13.0	6.9	13.7	0.5	8.6	−3.3	7.8	−3.4	54.187	<0.001	0.190
		Melatonin	12.2	12.2	1.6	10.6	−1.1	11.9	−0.2	11.9	−0.2	2.843	0.416	0.043
		Magnesium	12.1	12.1	−2.9	12.7	0.4	15.3	2.5	11.6	−0.3	11.068	0.011	0.086
		Citrul. M.	11.6	11.6	5.9	10.9	−0.5	11.9	0.2	3.9	−5.3	48.846	<0.001	0.180
		Glutamine	11.0	11.0	2.2	9.3	−1.2	13.1	1.7	7.2	−2.6	12.108	0.007	0.090

CG: Carbohydrate gainers; CM: Creatine monohydrate; BCAA: Branched-Chain Amino Acids; MC: Mineral complex; Citrul. M: Citrulline Malate.

## Data Availability

In alignment with Research Data Policies promoting open science, the dataset from this study is accessible to interested researchers via the Figshare repository: doi:10.6084/m9.figshare.28400753, accessed on 13 February 2025.

## Abbreviations

The following abbreviations are used in this manuscript:

AIS	Australian Institute of Sport
SS	Sports Supplement
